# Supplementary material for: Drug repurposing for aging research using model organisms
Source: Aging Cell. 2017 Jun 16;16(5):1006–15. doi: 10.1111/acel.12626 (PMC5595691; doi:10.1111/acel.12626)
Supplement: Supplementary file 7 — Data S1 Zip‐Archive of all report cards. [file ACEL-16-1006-s007.zip › RC_STU.pdf]

STU

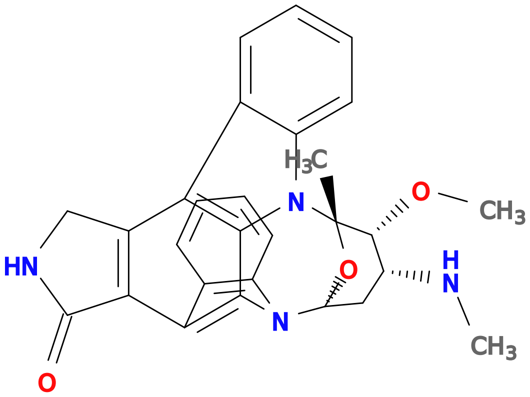

Database identifiers

ChEMBLCompound	CHEMBL388978
CHEBI	15738
eMolecules	30154559
eMolecules	715670

Ranking

	Rank	Score
Drosophila	191/697	0.711
C. elegans	481/591	0.089

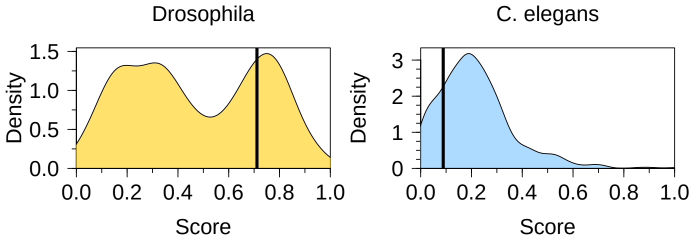

	Ageing implication	Domain conservation	Binding site conservation	Binding affinity	Bioavailability	Lipinski	Promiscuity	Purchasability	Drug approval	**Total**
Drosophila	1.0	0.973	1.0	0.979	(0.9)	-0.05	-0.196	0.1	0.0	0.711
C. elegans	1.0	0.97	1.0	0.979	0.248	-0.05	-0.196	0.1	0.0	0.089

Names

- **staurosporine**
- STS

Roles

EC 2.7.11.13 (protein kinase C) inhibitor

Status

Approved drug (according to ChEMBL)	No
Number of Rule of 5 violations	1
Binding affinity to original target in log units (RF-Score prediction)	8.87
Burns *C. elegans* bioavailability prediction	-2.21

Compound Target Characteristics

Serine/threonine-protein kinase Chk1

Best gene implication in ageing for this target family came from gene O14757 annotated in UniProt release 2014_02. Annotation GO subterm of 7568 (aging) was Non-traceable Author Statement

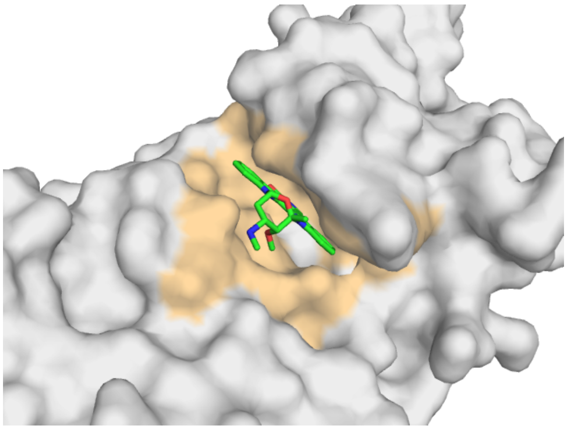

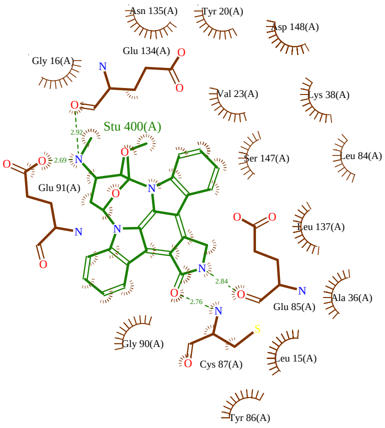

protein	amino acids contacts (binding site)															
PDB:1nvr:chainA:O14757	L	G	Y	V	A	K	L	E	Y	C	G	E	E	N	L	S	D
tr:E9PJI4:E9PJI4_HUMAN	L	G	Y	V	A	K	L	E	Y	C	G	E	E	N	L	S	D
tr:E9PKQ3:E9PKQ3_HUMAN	L	G	Y	V	A	K	L	E	Y	C	G	E	E	N	L	S	D
tr:J3KN87:J3KN87_HUMAN	L	G	Y	V	A	K	L	E	Y	C	G	E	E	N	L	S	D
sp:O14757:CHK1_HUMAN	L	G	Y	V	A	K	L	E	Y	C	G	E	E	N	L	S	D
sp:Q91ZN7:CHK1_RAT	L	G	Y	V	A	K	L	E	Y	C	G	E	E	N	L	S	D
tr:G3UYB1:G3UYB1_MOUSE	L	G	Y	V	A	K	L	E	Y	C	G	E	E	N	L	S	D
tr:G3UYC5:G3UYC5_MOUSE	L	G	Y	V	A	K	L	E	Y	C	G	E	E	N	L	S	D
sp:O35280:CHK1_MOUSE	L	G	Y	V	A	K	L	E	Y	C	G	E	E	N	L	S	D
sp:O61661:CHK1_DROME	L	G	Y	V	A	K	L	E	Y	A	G	E	E	N	L	S	D
sp:Q9N3Z3:CHK1_CAEEL	L	G	F	V	A	K	L	E	Y	A	G	E	E	N	L	S	D
sp:P38147:CHK1_YEAST	V	G	F	V	A	K	L	E	M	A	G	D	E	N	L	A	D

protein	whole protein ident	whole protein simil	domain-based ident	domain-based simil	contact-based ident	contact-based simil
PDB:1nvr:chainA:O14757	1.0	1.0	1.0	1.0	1.0	1.0
tr:E9PJI4:E9PJI4_HUMAN	0.36	0.36	0.63	0.63	1.0	1.0
tr:E9PKQ3:E9PKQ3_HUMAN	0.43	0.43	0.76	0.76	1.0	1.0
tr:J3KN87:J3KN87_HUMAN	0.91	0.91	1.0	1.0	1.0	1.0
sp:O14757:CHK1_HUMAN	1.0	1.0	1.0	1.0	1.0	1.0
sp:Q91ZN7:CHK1_RAT	0.94	0.98	0.98	1.0	1.0	1.0
tr:G3UYB1:G3UYB1_MOUSE	0.84	0.89	0.97	0.99	1.0	1.0
tr:G3UYC5:G3UYC5_MOUSE	0.87	0.91	0.97	0.99	1.0	1.0
sp:O35280:CHK1_MOUSE	0.93	0.98	0.97	0.99	1.0	1.0
sp:O61661:CHK1_DROME	0.44	0.75	0.56	0.87	0.94	0.89
sp:Q9N3Z3:CHK1_CAEEL	0.31	0.63	0.48	0.8	0.88	0.87
sp:P38147:CHK1_YEAST	0.22	0.59	0.35	0.74	0.65	0.75

grp (FBgn0261278) associated phenotypes

cell cycle defective, chemical sensitive, decreased cell death, maternal effect, mitotic cell cycle defective, non-rescuable maternal effect, partially lethal - majority die, radiation resistant, radiation

sensitive

(Information from FlyBase)

grp (UniProt:O61661) annotation
Function: Serine/threonine-protein kinase which is required for checkpoint-mediated cell cycle arrest and activation of DNA repair in response to the presence of DNA damage or unreplicated DNA. May also negatively regulate cell cycle progression during unperturbed cell cycles. May phosphorylate the CDC25 phosphatase stg, which promotes its degradation. This results in increased inhibitory tyrosine phosphorylation of CDC2-cyclin complexes and consequent inhibition of cell cycle progression. (PubMed:10209095, PubMed:10469601, PubMed:10980701, PubMed:12919679, PubMed:14711410, PubMed:15723794, PubMed:15860729, PubMed:16079276, PubMed:7925016, PubMed:9197245, PubMed:9214509).
Subcellular location: Nucleus ECO:0000269—PubMed:10980701, PubMed:16079276).
Developmental stage: Expressed both maternally and zygotically. Maternally supplied mRNA is degraded during progression from nuclear stage 12 to nuclear stage 13. Zygotic expression is seen at reduced levels later in embryogenesis and during larval development. Higher expression is seen in pupae, coincident with ovarian differentiation. May be activated during the syncytial blastoderm divisions which precede cellularization, the Drosophila equivalent of the midblastula transition (MBT). Developmentally regulated activation of the DNA replication checkpoint may occur as the nucleo-cytoplasmic ratio increases and maternal replication factors are depleted. Elongation of the embryonic cell cycle may allow time for the transcription of genes that initiate the switch from maternal to zygotic control of embryogenesis. (PubMed:9197245, PubMed:9214509).
Ptm: Phosphorylated in a MEI-41/ATR dependent manner in response to DNA damage or the presence of unreplicated DNA. (PubMed:15860729).

(Information from UniProt)

chk-1 (WBGene00000498) associated phenotypes
lethal, locomotion variant, mid larval lethal, sterile

(Information from WormBase)

chk-1 (UniProt:Q9N3Z3) annotation
Function: Serine/threonine-protein kinase which is required for checkpoint-mediated cell cycle arrest and activation of DNA repair in response to the presence of DNA damage or unreplicated DNA. May also negatively regulate cell cycle progression during unperturbed cell cycles. Required for checkpoint mediated cell cycle arrest in response to DNA damage in germline cells. Essential for embryogenesis. (PubMed:15326393).
Subcellular location: Cytoplasm Nucleus
Tissue specificity: Expressed in the germline. (PubMed:15326393).
Developmental stage: Highly expressed in the embryo. (PubMed:15326393).

(Information from UniProt)

3-phosphoinositide-dependent protein kinase 1

Best gene implication in ageing for this target family came from gene Q9Y1J3 via mapping the annotation from Ensembl WBGene00003965 via mapping the annotation from EntrezGene 180475 via mapping the annotation from GenAgeModels 0499 annotated in GenAge release 17.

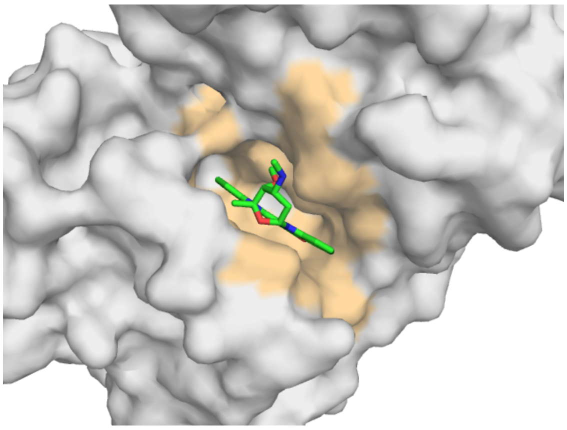

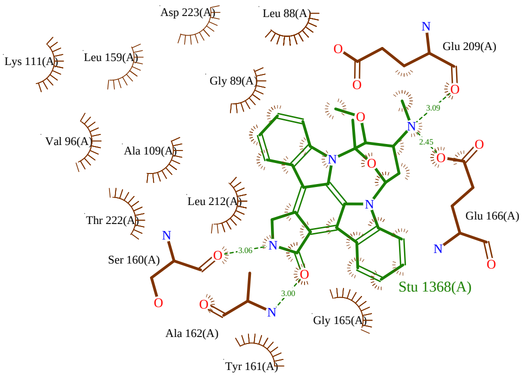

protein	amino acids contacts (binding site)
PDB:1oky:chainA:O15530	L G V A K L S Y A G E E L T D
tr:Q9UPJ8:Q9UPJ8_HUMAN	L G V A K L S Y A G E E L T D
tr:E9PER6:E9PER6_HUMAN	L G V A K L S Y A G E E L T D
tr:C9JWR9:C9JWR9_HUMAN	L G V A K L S Y A G E E L T D
sp:O15530:PDPK1_HUMAN	L G V A K L S Y A G E E L T D
sp:O55173:PDPK1_RAT	L G V A K L S Y A G E E L T D
tr:Q3UEW8:Q3UEW8_MOUSE	L G V A K L S Y A G E E L T D
tr:F2Z400:F2Z400_MOUSE	L G V A K L S Y A G E E L T D
tr:Q3TRL2:Q3TRL2_MOUSE	L G V A K L S Y A G E E L T D
tr:F2Z3X6:F2Z3X6_MOUSE	L G V A K L S Y A G E E L T D
sp:Q9Z2A0:PDPK1_MOUSE	L G V A K L S Y A G E E L T D
tr:Q810Z4:Q810Z4_MOUSE	L G V A K L S Y A G E E L T D
tr:A4V164:A4V164_DROME	I G V A K M T Y A G D E L A D
tr:C7LAC8:C7LAC8_DROME	I G V A K M T Y A G D E L A D
tr:H5V8B9:H5V8B9_DROME	I G V A K M T Y A G D E L A D
sp:Q9W0V1:PDPK1_DROME	I G V A K M T Y A G D E L A D
sp:Q9Y1J3:PDPK1_CAEEL	M G V A K I G L V G D D L T D
sp:Q03407:PKH1_YEAST	L G V A K L E Y A G D E L T D
sp:Q03306:PKH3_YEAST	L G V A K L D F A G E E L T D

	whole protein		domain-based		contact-based	
protein	ident	simil	ident	simil	ident	simil
PDB:1oky:chainA:O15530	1.0	1.0	1.0	1.0	1.0	1.0
tr:Q9UPJ8:Q9UPJ8_HUMAN	0.66	0.66	1.0	1.0	1.0	1.0
tr:E9PER6:E9PER6_HUMAN	0.95	0.95	1.0	1.0	1.0	1.0
tr:C9JWR9:C9JWR9_HUMAN	0.95	0.96	0.91	0.91	1.0	1.0
sp:O15530:PDPK1_HUMAN	1.0	1.0	1.0	1.0	1.0	1.0
sp:O55173:PDPK1_RAT	0.95	0.98	0.99	1.0	1.0	1.0
tr:Q3UEW8:Q3UEW8_MOUSE	0.73	0.76	0.99	1.0	1.0	1.0
tr:F2Z400:F2Z400_MOUSE	0.75	0.79	0.99	1.0	1.0	1.0
tr:Q3TRL2:Q3TRL2_MOUSE	0.88	0.91	0.99	1.0	1.0	1.0
tr:F2Z3X6:F2Z3X6_MOUSE	0.9	0.93	0.99	1.0	1.0	1.0
sp:Q9Z2A0:PDPK1_MOUSE	0.95	0.98	0.99	1.0	1.0	1.0
tr:Q810Z4:Q810Z4_MOUSE	0.93	0.97	0.99	1.0	1.0	1.0
tr:A4V164:A4V164_DROME	0.31	0.56	0.43	0.68	0.67	0.88
tr:C7LAC8:C7LAC8_DROME	0.28	0.5	0.43	0.68	0.67	0.88
tr:H5V8B9:H5V8B9_DROME	0.28	0.5	0.43	0.68	0.67	0.88
sp:Q9W0V1:PDPK1_DROME	0.28	0.5	0.43	0.68	0.67	0.88
sp:Q9Y1J3:PDPK1_CAEEL	0.28	0.59	0.33	0.66	0.53	0.82
sp:Q03407:PKH1_YEAST	0.21	0.47	0.44	0.74	0.87	0.92
sp:Q03306:PKH3_YEAST	0.15	0.35	0.4	0.69	0.87	0.94

Pdk1 (FBgn0020386) associated phenotypes

cell autonomous, chemical conditional, chemical resistant, chemical sensitive, decreased cell size, developmental rate defective, increased cell death, increased cell size, male limited, neuroanatomy defective, nutrition conditional, partially lethal - majority die, small body, somatic clone

(Information from FlyBase)

Pdk1 (UniProt:Q9W0V1) annotation
Function: Serine/threonine kinase required for embryonic development. Inhibits apoptosis. Acts in the insulin receptor transduction pathway which regulates cell growth and organ size, by phosphorylating and activating Akt1 and S6k. May be involved in axonal pathfinding and synaptogenesis, and in spermatogenesis. (PubMed:10336630, PubMed:11301252, PubMed:11344272, PubMed:11752451, PubMed:11862217, PubMed:15238523, PubMed:9368760).
Subcellular location: Cytoplasm
Developmental stage: Highly expressed in Malpighian tubule primordia from stage late 11 to 13. Expressed in hindgut at stage 13. After stage 14, ubiquitously expressed at low levels. (PubMed:11301252).
Domain: The PIF-pocket is a small lobe in the catalytic domain required by the enzyme for the binding to the hydrophobic motif of its substrates. It is an allosteric regulatory site that can accommodate small compounds acting as allosteric inhibitors. (UniProtKB:O15530).
Disruption phenotype: Death during the second instar stage. (PubMed:11752451).
(Information from UniProt)

pdk-1 (WBGene00003965) associated phenotypes
DMPP resistant, carbon dioxide avoidance variant, dauer constitutive, dauer recovery inhibited, egg laying variant, extended life span, long, reduced brood size, shortened life span, social feeding increased
(Information from WormBase)

pdk-1 (UniProt:Q9Y1J3) annotation
Function: Involved in the daf-2/insulin receptor-like transduction pathway, which controls longevity and prevents developmental arrest at the dauer stage. Phosphorylates and activates sgk-1, akt-1 and akt-2. (PubMed:10364160, PubMed:15068796).
Subunit: Interacts directly with sgk-1, akt-1 and akt-2.(PubMed:15068796).
Subcellular location: Cytoplasm
Developmental stage: Expressed in late stage embryos and throughout life. At L1, expressed in neurons, intestinal cells and hypodermal cells. In adults, expressed in the somatic gonad. (PubMed:10364160).
Domain: The PIF-pocket is a small lobe in the catalytic domain required by the enzyme for the binding to the hydrophobic motif of its substrates. It is an allosteric regulatory site that can accommodate small compounds acting as allosteric inhibitors. (UniProtKB:O15530).
(Information from UniProt)

Ribosomal protein S6 kinase beta-1

Best gene implication in ageing for this target family came from gene Q8SWT5 annotated in UniProt release 2014_02. Annotation GO 8340 (determination of adult lifespan) was Inferred from Mutant Phenotype

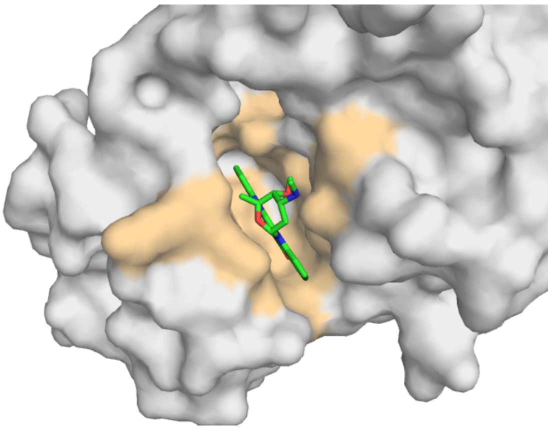

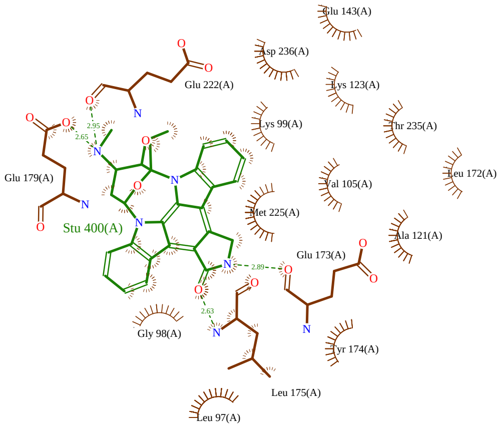

protein	amino acids contacts (binding site)																	
PDB:3a60:chainA:P23443	L	G	K	G	V	A	K	E	L	E	Y	L	E	E	N	M	T	D
PDB:3a61:chainA:P23443	L	G	K	G	V	A	K	E	L	E	Y	L	E	E	N	M	T	D
PDB:3a62:chainA:P23443	L	G	K	G	V	A	K	E	L	E	Y	L	E	E	N	M	T	D
sp:P23443:KS6B1_HUMAN	L	G	K	G	V	A	K	E	L	E	Y	L	E	E	N	M	T	D
sp:P67999:KS6B1_RAT	L	G	K	G	V	A	K	E	L	E	Y	L	E	E	N	M	T	D
sp:Q8BSK8:KS6B1_MOUSE	L	G	K	G	V	A	K	E	L	E	Y	L	E	E	N	M	T	D
tr:Q8SWT5:Q8SWT5_DROME	L	G	K	G	V	A	K	E	L	E	Y	L	E	E	N	L	T	D
tr:H1ZYE3:H1ZYE3_DROME	L	G	K	G	V	A	K	E	L	E	Y	L	E	E	N	L	T	D
tr:Q94533:Q94533_DROME	L	G	K	G	V	A	K	E	L	E	Y	L	E	E	N	L	T	D
tr:P91656:P91656_DROME	L	G	K	G	V	A	K	E	L	E	Y	L	E	E	N	L	T	D
tr:Q9NAH6:Q9NAH6_CAEEL	L	G	K	G	V	A	K	E	L	E	Y	L	E	E	N	L	T	D
sp:P11792:SCH9_YEAST	L	G	K	G	V	A	K	E	T	D	Y	M	E	E	N	L	C	D

	whole protein		domain-based		contact-based	
protein	ident	simil	ident	simil	ident	simil
PDB:3a60:chainA:P23443	1.0	1.0	1.0	1.0	1.0	1.0
PDB:3a61:chainA:P23443	1.0	1.0	1.0	1.0	1.0	1.0
PDB:3a62:chainA:P23443	1.0	1.0	1.0	1.0	1.0	1.0
sp:P23443:KS6B1_HUMAN	1.0	1.0	1.0	1.0	1.0	1.0
sp:P67999:KS6B1_RAT	1.0	1.0	1.0	1.0	1.0	1.0
sp:Q8BSK8:KS6B1_MOUSE	0.99	1.0	1.0	1.0	1.0	1.0
tr:Q8SWT5:Q8SWT5_DROME	0.28	0.38	0.5	0.6	0.94	0.99
tr:H1ZYE3:H1ZYE3_DROME	0.49	0.64	0.76	0.93	0.94	0.99
tr:Q94533:Q94533_DROME	0.5	0.72	0.76	0.93	0.94	0.99
tr:P91656:P91656_DROME	0.41	0.61	0.76	0.93	0.94	0.99
tr:Q9NAH6:Q9NAH6_CAEEL	0.46	0.72	0.74	0.92	0.94	0.99
sp:P11792:SCH9_YEAST	0.21	0.44	0.5	0.81	0.72	0.84

S6k (FBgn0015806) associated phenotypes

cell autonomous, chemical resistant, decreased cell number, decreased cell size, feeding behavior defective, heat sensitive, increased cell growth, increased cell number, increased cell size, large body, long lived, memory defective, neuroanatomy defective, neurophysiology defective, nutrition conditional, partially, partially lethal - majority die, short lived, size defective, small body, somatic clone

(Information from FlyBase)

rsks-1 (WBGene00012929) associated phenotypes

aging variant, extended life span, fertility reduced, lethal, progressive paralysis, slow development, small, sterile, swimming variant, translation variant

(Information from WormBase)

Tyrosine-protein kinase ZAP-70

Best gene implication in ageing for this target family came from gene Q24592 via mapping the annotation from Ensembl FBgn0004864 via mapping the annotation from EntrezGene 32080 via mapping the annotation from GenAgeModels 1818 annotated in GenAge release 17.

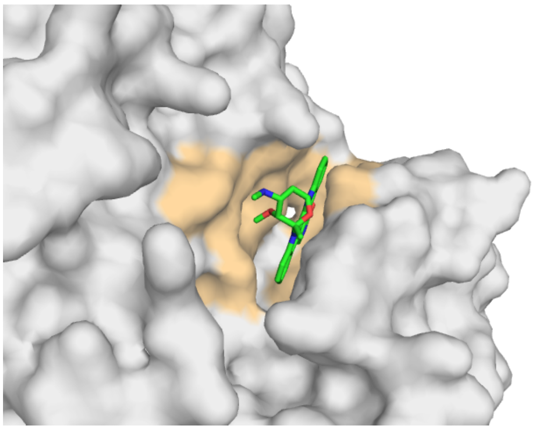

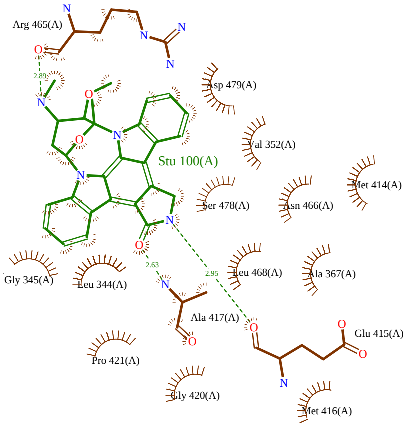

protein	amino acids contacts (binding site)
PDB:1u59:chainA:P43403	L G V A M E M A G P R N L S D
sp:P43403:ZAP70_HUMAN	L G V A M E M A G P R N L S D
tr:D3ZH44:D3ZH44_RAT	L G V A M E M A G P R N L S D
tr:Q63614:Q63614_RAT	L G V A M E M A G P R N L S D
tr:G3V7N4:G3V7N4_RAT	L G V A M E M A G P R N L S D
tr:E9PWE9:E9PWE9_MOUSE	L G V A M E M A G P R N L S D
tr:Q3UPF7:Q3UPF7_MOUSE	L G V A M E M A G P R N L S D
sp:P48025:KSYK_MOUSE	L G V A M E M A G P R N L S D
sp:Q24592:JAK_DROME	I G V A M E Y L G S R N L S D

	whole protein		domain-based		contact-based	
protein	ident	simil	ident	simil	ident	simil
PDB:1u59:chainA:P43403	0.98	0.99	1.0	1.0	1.0	1.0
sp:P43403:ZAP70_HUMAN	1.0	1.0	1.0	1.0	1.0	1.0
tr:D3ZH44:D3ZH44_RAT	0.53	0.82	0.65	0.88	1.0	1.0
tr:Q63614:Q63614_RAT	0.51	0.79	0.65	0.88	1.0	1.0
tr:G3V7N4:G3V7N4_RAT	0.51	0.8	0.65	0.88	1.0	1.0
tr:E9PWE9:E9PWE9_MOUSE	0.51	0.79	0.63	0.85	1.0	1.0
tr:Q3UPF7:Q3UPF7_MOUSE	0.51	0.8	0.64	0.88	1.0	1.0
sp:P48025:KSYK_MOUSE	0.51	0.8	0.64	0.88	1.0	1.0
sp:Q24592:JAK_DROME	0.1	0.31	0.33	0.73	0.73	0.86

hop (FBgn0004864) associated phenotypes

RU486 conditional, cell autonomous, cell non-autonomous, cell shape defective, dominant, germline clone, heat sensitive, hyperplasia, immune response defective, increased cell number, maternal effect, melanotic mass phenotype, memory defective, neuroanatomy defective, partially, partially lethal - majority die, planar polarity defective, polyphasic, reduced, rescuable maternal effect, somatic clone, tumorigenic

(Information from FlyBase)

hop (UniProt:Q24592) annotation

Function: Tyrosine kinase of the non-receptor type, phosphorylates the marelle protein. Required maternally for the establishment of the normal array of embryonic segments: involved in the control of pair-rule gene transcription in a stripe-specific manner. Together with Hsp83 and piwi, mediates canalization, also known as developmental robustness, likely via epigenetic silencing of existing genetic variants and suppression of transposon-induced new genetic variation. (PubMed:21186352, PubMed:8314084).

Subunit: Forms a complex with Hsp83 and piwi; probably Hop mediates the interaction between

piwi and Hsp83.

Subcellular location: Endomembrane system ; Peripheral membrane protein Note=Wholly intracellular, possibly membrane associated.

Developmental stage: Expressed both maternally and zygotically throughout development. (PubMed:8314084).

Domain: Possesses two phosphotransferase domains. The second one probably contains the catalytic domain (By similarity), while the presence of slight differences suggest a different role for domain 1 (By similarity).

(Information from UniProt)

Glycogen synthase kinase-3 beta

Best gene implication in ageing for this target family came from gene P49841 via mapping the annotation from Ensembl ENSG00000082701 via mapping the annotation from EntrezGene 2932 via mapping the annotation from GenAgeHuman 0097 annotated in GenAge release 17.

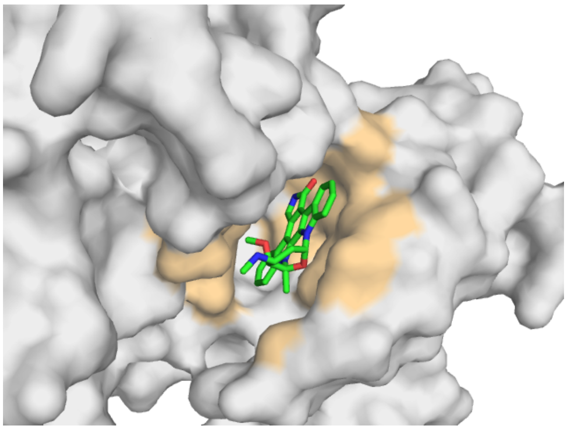

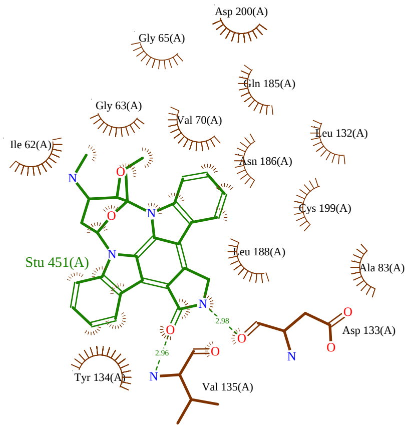

protein	amino acids contacts (binding site)
PDB:1q3d:chainA:P49841	I G G V A L D Y V Q N L C D
tr:Q6FI27:Q6FI27_HUMAN	I G G V A L D Y V Q N L C D
sp:P49841:GSK3B_HUMAN	I G G V A L D Y V Q N L C D
sp:P18266:GSK3B_RAT	I G G V A L D Y V Q N L C D
tr:Q5KU03:Q5KU03_MOUSE	I G G V A L D Y V Q N L C D
sp:Q9WV60:GSK3B_MOUSE	I G G V A L D Y V Q N L C D
tr:E9QAQ5:E9QAQ5_MOUSE	I G G V A L D Y V Q N L C D
tr:B6IDN4:B6IDN4_DROME	I G G V A L E Y I Q N L C D
sp:P18431:SGG_DROME	I G G V A L E Y I Q N L C D
tr:A4V3W2:A4V3W2_DROME	I G G V A L E Y I Q N L C D
tr:A4V3W1:A4V3W1_DROME	I G G V A L E Y I Q N L C D
tr:Q0KHW6:Q0KHW6_DROME	I G G V A L E Y I Q N L C D
tr:E6PBX9:E6PBX9_DROME	I G G V A L E Y I Q N L C D
tr:H5V852:H5V852_DROME	I G G V A L E Y I Q N L C D
sp:Q9U2Q9:GSK3_CAEEL	I G G V A L E Y V Q N L C D
sp:P50873:MRK1_YEAST	V G G V A L D Y M Q N L C D

protein	whole protein ident	whole protein simil	domain-based ident	domain-based simil	contact-based ident	contact-based simil
PDB:1q3d:chainA:P49841	1.0	1.0	1.0	1.0	1.0	1.0
tr:Q6FI27:Q6FI27_HUMAN	1.0	1.0	1.0	1.0	1.0	1.0
sp:P49841:GSK3B_HUMAN	1.0	1.0	1.0	1.0	1.0	1.0
sp:P18266:GSK3B_RAT	0.99	1.0	1.0	1.0	1.0	1.0
tr:Q5KU03:Q5KU03_MOUSE	0.99	1.0	1.0	1.0	1.0	1.0
sp:Q9WV60:GSK3B_MOUSE	0.99	1.0	1.0	1.0	1.0	1.0
tr:E9QAQ5:E9QAQ5_MOUSE	0.96	0.97	0.96	0.96	1.0	1.0
tr:B6IDN4:B6IDN4_DROME	0.58	0.71	0.85	0.96	0.86	0.95
sp:P18431:SGG_DROME	0.59	0.74	0.85	0.96	0.86	0.95
tr:A4V3W2:A4V3W2_DROME	0.59	0.74	0.85	0.96	0.86	0.95
tr:A4V3W1:A4V3W1_DROME	0.52	0.66	0.85	0.96	0.86	0.95
tr:Q0KHW6:Q0KHW6_DROME	0.49	0.63	0.85	0.96	0.86	0.95
tr:E6PBX9:E6PBX9_DROME	0.18	0.26	0.52	0.65	0.86	0.95
tr:H5V852:H5V852_DROME	0.28	0.35	0.85	0.96	0.86	0.95
sp:Q9U2Q9:GSK3_CAEEL	0.61	0.76	0.81	0.95	0.93	0.97
sp:P50873:MRK1_YEAST	0.31	0.57	0.53	0.84	0.86	0.97

sgg (FBgn0003371) associated phenotypes

cell non-autonomous, cell polarity defective, chemical sensitive, circadian rhythm defective, developmental rate defective, germline clone, heat sensitive, lethal - all die before end of P-stage, lethal - all die before end of pupal stage, maternal effect, meiotic cell cycle defective, mitotic cell cycle defective, neuroanatomy defective, neurophysiology defective, non-rescuable maternal effect, planar polarity defective, rescuable maternal effect, short lived, smell perception defective, somatic clone, some die during pharate adult stage, some die during pupal stage, some die during third instar larval stage

(Information from FlyBase)

sgg (UniProt:P18431) annotation

Function: Required for several developmental events such as syncytial blastoderm formation and embryonic segmentation. Is involved in transcriptional regulation. Required for arm phosphorylation. Wg signaling operates by inactivating the sgg repression of en autoactivation. Negatively controls the neuromuscular junction (NMJ) growth in presynaptic motoneurons. Plays a role in the regulation of microtubule dynamics and actin cytoskeleton during embryogenesis. Required for phosphorylation of sra in activated eggs. Essential for completion of meiosis, possibly by triggering calcineurin activation via sra phosphorylation. Phosphorylates microtubule-associated protein futsch in axons. (PubMed:15269269, PubMed:16570248, PubMed:16949836, PubMed:2113617, PubMed:2118107, PubMed:22421435, PubMed:7529201, PubMed:8467811).
Subunit: Interacts with cos. (PubMed:1335365,PubMed:15691767).
Subcellular location: Cytoplasm. Nucleus. Cytoplasm, cytoskeleton, microtubule organizing center, centrosome. Cytoplasm, cell cortex. Cell junction, synapse. Cell projection, axon. Note=In syncytial embryos, detected at the centrosomes throughout the cell cycle, and in the mitotic spindle and pseudocleavage furrows invaginating from the cell cortex during mitosis. Concentrated at the growing end of membranes during the cellularization process. After cellularization, localized to the centrosomes during mitosis and to the nucleus at the end of telophase. Enriched in the presynaptic side of the neuromuscular junction, with some signal detected also in axonal branches.
Tissue specificity: Expressed in ovaries and activated eggs (at protein level). Expression is over all the embryo at all stages, no local accumulation is observed. (PubMed:2118107, PubMed:22421435, PubMed:8467811).
Developmental stage: Isoform SGG46 is expressed at low levels in 12-24 hours embryos. Isoform Zygotic and isoform SGG39 are expressed in 12-24 hours embryos and present throughout the larval, pupal and adult stages (at protein level). Isoform Zygotic is expressed maternally and zygotically but reduced throughout later embryonic development. Expression persists throughout larval stages. (PubMed:2118107, PubMed:8467811).
Disruption phenotype: Mutants display an overdeveloped neuromuscular junction (NMJ), with the number of boutons greatly increased. (PubMed:15269269).

(Information from UniProt)

gsk-3 (WBGene00001746) associated phenotypes
axon morphology variant, larval lethal, lethal, locomotion variant, sterile
(Information from WormBase)

gsk-3 (UniProt:Q9U2Q9) annotation
Function: Phosphorylates oma-1, a regulator of the oocyte-to- embryo transition, enabling its degradation. Phosphorylates skn-1, preventing it from accumulating in nuclei and thus inhibiting phase II gene expression in the oxidative stress defense. Involved in mesendoderm specification and mitotic spindle orientation in EMS blastomeres. Thought to be a branch point in these processes as proteins downstream are not required. Negatively regulates Wnt signaling in vulval precursor cells and acts as a Wnt-independent repressor of med-1 and med-2 in the C lineage inhibiting mesoderm development. Required for normal lifespan and LiCl-induced lifespan extension. (PubMed:10444600, PubMed:11463373, PubMed:15572126, PubMed:16251270, PubMed:16289132, PubMed:16343905, PubMed:16930586, PubMed:17959600).
Subunit: Monomer (By similarity). Interacts with axl-1. (UniProtKB:Q9WV60, PubMed:17601533).
Disruption phenotype: Worms exhibit defects in endoderm specification and mitotic spindle alignment. Mutants show reduced degradation of oma-1 and have shortened lifespan. (PubMed:10444600).
(Information from UniProt)

5'-AMP-activated protein kinase catalytic subunit alpha-1

Best gene implication in ageing for this target family came from gene Q95ZQ4 annotated in UniProt release 2014_02. Annotation GO 8340 (determination of adult lifespan) was Inferred from Mutant Phenotype

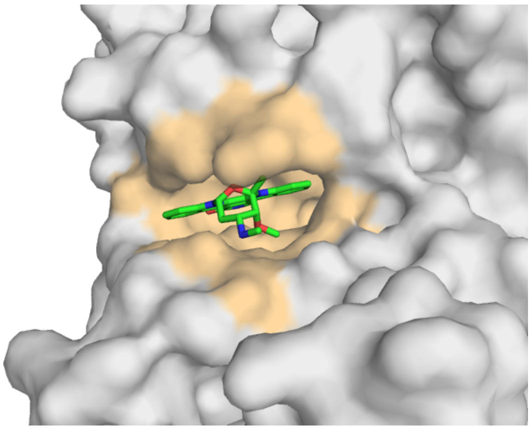

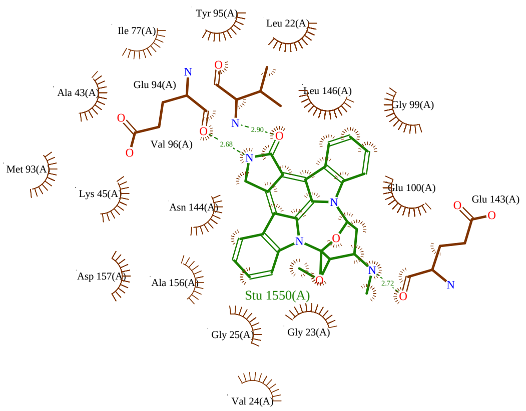

protein	amino acids contacts (binding site)
PDB:4cfh:chainA:P54645	L G V G A K I M E Y V G E E N L A D
tr:Q96E92:Q96E92_HUMAN	L G V G A K I M E Y V G E E N L A D
sp:P54646:AAPK2_HUMAN	L G V G A K I M E Y V G E E N L A D
sp:Q13131:AAPK1_HUMAN	L G V G A K I M E Y V G E E N L A D
tr:G3V715:G3V715_RAT	L G V G A K I M E Y V G E E N L A D
sp:P54645:AAPK1_RAT	L G V G A K I M E Y V G E E N L A D
sp:Q5EG47:AAPK1_MOUSE	L G V G A K I M E Y V G E E N L A D
tr:Q3TUQ7:Q3TUQ7_MOUSE	L G V G A K I M E Y V G E E N L A D
sp:Q8BRK8:AAPK2_MOUSE	L G V G A K I M E Y V G E E N L A D
tr:Q5U118:Q5U118_DROME	L G T G A K I M E Y V G E E N L A D
tr:O18645:O18645_DROME	L G T G A K I M E Y V G E E N L A D
sp:Q95ZQ4:AAPK2_CAEEL	L G V G A K I M E H V G E E N L A D
sp:P06782:SNF1_YEAST	L G E G A K I I E Y - N E E N L A D

	whole protein		domain-based		contact-based	
protein	ident	simil	ident	simil	ident	simil
PDB:4cfh:chainA:P54645	1.0	1.0	1.0	1.0	1.0	1.0
tr:Q96E92:Q96E92_HUMAN	0.34	0.36	0.7	0.71	1.0	1.0
sp:P54646:AAPK2_HUMAN	0.74	0.89	0.91	0.97	1.0	1.0
sp:Q13131:AAPK1_HUMAN	0.99	1.0	1.0	1.0	1.0	1.0
tr:G3V715:G3V715_RAT	0.74	0.89	0.9	0.97	1.0	1.0
sp:P54645:AAPK1_RAT	1.0	1.0	1.0	1.0	1.0	1.0
sp:Q5EG47:AAPK1_MOUSE	1.0	1.0	1.0	1.0	1.0	1.0
tr:Q3TUQ7:Q3TUQ7_MOUSE	0.98	0.98	1.0	1.0	1.0	1.0
sp:Q8BRK8:AAPK2_MOUSE	0.74	0.89	0.9	0.97	1.0	1.0
tr:Q5U118:Q5U118_DROME	0.53	0.75	0.78	0.93	0.94	0.96
tr:O18645:O18645_DROME	0.54	0.75	0.78	0.93	0.94	0.96
sp:Q95ZQ4:AAPK2_CAEEL	0.5	0.73	0.78	0.93	0.94	0.96
sp:P06782:SNF1_YEAST	0.31	0.58	0.58	0.86	0.78	0.78

SNF1A (FBgn0023169) associated phenotypes

RU486 conditional, cell cycle defective, cell polarity defective, cytokinesis defective, developmental rate defective, dominant, feeding behavior defective, hyperactive, immune response defective, increased cell size, lethal - all die before end of second instar larval stage, long lived, maternal effect, mitotic cell cycle defective, neuroanatomy defective, nutrition conditional, short lived, small body, somatic clone, some die during second instar larval stage, some die during third instar larval stage, starvation stress response defective, stress response defective

(Information from FlyBase)

aak-2 (WBGene00020142) associated phenotypes

drug induced gene expression variant, drug induced life span variant, drug induced locomotion variant, drug response variant, frequency body bend reduced, organism oxidative stress response hypersensitive, paraquat hypersensitive, protein phosphorylation reduced, suppression of head oscillations defective

(Information from WormBase)

aak-2 (UniProt:Q95ZQ4) annotation

Function: Acts as a sensor that couples lifespan to information about energy levels and insulin-like signals. Role in motility and response to oxidative stress. Causes germ cell cycle arrest upon dauer entry. (PubMed:15574588, PubMed:18408008).
Enzyme regulation: Activated by phosphorylation.(PubMed:15574588).
Subunit: Tetramer, composed of 2 regulatory (R) and 2 catalytic (C) subunits. In the presence of cAMP it dissociates into 2 active monomeric C subunits and an R dimer that binds four cAMP molecules. (PubMed:2303451).
Tissue specificity: Expressed in the pharynx, the ventral cord, neurons including the hermaphrodite-specific neuron, body wall muscles, the vulva, the excretory canal, and weakly in the intestine. (PubMed:18408008).

Ptm: Phosphorylated on Thr-243 in response to oxidative stress. (PubMed:15574588).
Disruption phenotype: Shortened life-span. Temperature-dependent hypersensitivity to stress, slow body bending, abnormal modulation of head oscillation. (PubMed:15574588, PubMed:18408008).

(Information from UniProt)

5'-AMP-activated protein kinase catalytic subunit alpha-2

Best gene implication in ageing for this target family came from gene Q95ZQ4 annotated in UniProt release 2014_02. Annotation GO 8340 (determination of adult lifespan) was Inferred from Mutant Phenotype

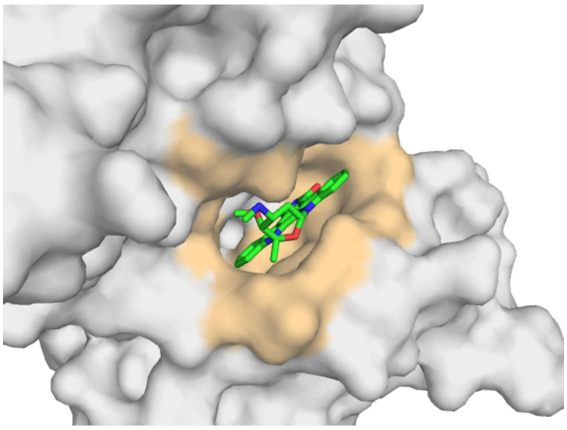

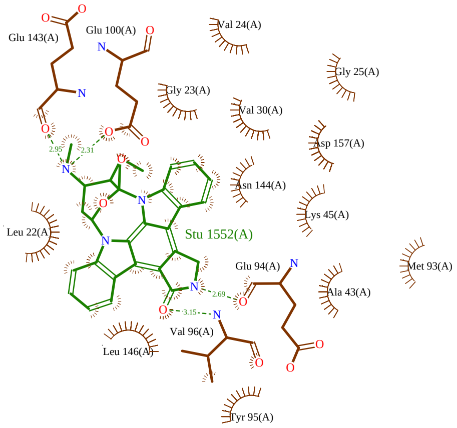

protein	amino acids contacts (binding site)																				
PDB:4cfe:chainA:P54646	L	G	V	G	V	A	K	M	E	Y	V	E	R	E	N	L	A	D	P	S	R
PDB:4cfe:chainC:P54646	L	G	V	G	V	A	K	M	E	Y	V	E	R	E	N	L	A	D	P	S	R
PDB:4cff:chainA:P54646	L	G	V	G	V	A	K	M	E	Y	V	E	R	E	N	L	A	D	P	S	R
sp:P54646:AAPK2_HUMAN	L	G	V	G	V	A	K	M	E	Y	V	E	R	E	N	L	A	D	P	S	R
tr:G3V715:G3V715_RAT	L	G	V	G	V	A	K	M	E	Y	V	E	R	E	N	L	A	D	P	S	R
sp:Q8BRK8:AAPK2_MOUSE	L	G	V	G	V	A	K	M	E	Y	V	E	R	E	N	L	A	D	P	S	R
tr:Q5U118:Q5U118_DROME	L	G	T	G	V	A	K	M	E	Y	V	E	R	E	N	L	A	D	P	S	K
tr:O18645:O18645_DROME	L	G	T	G	V	A	K	M	E	Y	V	E	R	E	N	L	A	D	P	S	K
sp:Q95ZQ4:AAPK2_CAEEL	L	G	V	G	V	A	K	M	E	H	V	E	R	E	N	L	A	D	P	S	K
sp:P06782:SNF1_YEAST	L	G	E	G	V	A	K	I	E	Y	-	E	R	E	N	L	A	D	P	S	S

	whole protein		domain-based		contact-based	
protein	ident	simil	ident	simil	ident	simil
PDB:4cfe:chainA:P54646	1.0	1.0	1.0	1.0	1.0	1.0
PDB:4cfe:chainC:P54646	1.0	1.0	1.0	1.0	1.0	1.0
PDB:4cff:chainA:P54646	1.0	1.0	1.0	1.0	1.0	1.0
sp:P54646:AAPK2_HUMAN	1.0	1.0	1.0	1.0	1.0	1.0
tr:G3V715:G3V715_RAT	0.98	1.0	0.99	1.0	1.0	1.0
sp:Q8BRK8:AAPK2_MOUSE	0.98	0.99	0.99	1.0	1.0	1.0
tr:Q5U118:Q5U118_DROME	0.57	0.76	0.8	0.94	0.9	0.96
tr:O18645:O18645_DROME	0.57	0.76	0.8	0.94	0.9	0.96
sp:Q95ZQ4:AAPK2_CAEEL	0.5	0.71	0.76	0.93	0.9	0.95
sp:P06782:SNF1_YEAST	0.33	0.59	0.59	0.86	0.81	0.79

SNF1A (FBgn0023169) associated phenotypes

RU486 conditional, cell cycle defective, cell polarity defective, cytokinesis defective, developmental rate defective, dominant, feeding behavior defective, hyperactive, immune response defective, increased cell size, lethal - all die before end of second instar larval stage, long lived, maternal effect, mitotic cell cycle defective, neuroanatomy defective, nutrition conditional, short lived, small body, somatic clone, some die during second instar larval stage, some die during third instar larval stage, starvation stress response defective, stress response defective

(Information from FlyBase)

aak-2 (WBGene00020142) associated phenotypes

drug induced gene expression variant, drug induced life span variant, drug induced locomotion variant, drug response variant, frequency body bend reduced, organism oxidative stress response hypersensitive, paraquat hypersensitive, protein phosphorylation reduced, suppression of head oscillations defective

(Information from WormBase)

aak-2 (UniProt:Q95ZQ4) annotation

Function: Acts as a sensor that couples lifespan to information about energy levels and insulin-like signals. Role in motility and response to oxidative stress. Causes germ cell cycle arrest upon dauer entry. (PubMed:15574588, PubMed:18408008).
Enzyme regulation: Activated by phosphorylation.(PubMed:15574588).
Subunit: Tetramer, composed of 2 regulatory (R) and 2 catalytic (C) subunits. In the presence of cAMP it dissociates into 2 active monomeric C subunits and an R dimer that binds four cAMP molecules. (PubMed:2303451).
Tissue specificity: Expressed in the pharynx, the ventral cord, neurons including the hermaphrodite-specific neuron, body wall muscles, the vulva, the excretory canal, and weakly in the intestine. (PubMed:18408008).
Ptm: Phosphorylated on Thr-243 in response to oxidative stress. (PubMed:15574588).
Disruption phenotype: Shortened life-span. Temperature-dependent hypersensitivity to stress, slow body bending, abnormal modulation of head oscillation. (PubMed:15574588, PubMed:18408008).

(Information from UniProt)

Protein kinase C theta type

Best gene implication in ageing for this target family came from gene P34722 via mapping the annotation from WormBase WBGene00006599 annotated in WormBase 2014-03-11. Annotation GO 8340 (determination of adult lifespan) was Inferred from Mutant Phenotype

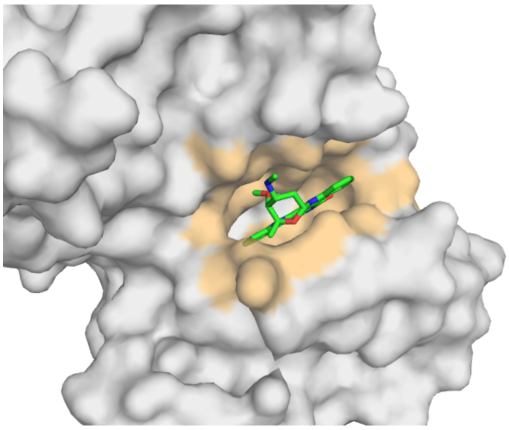

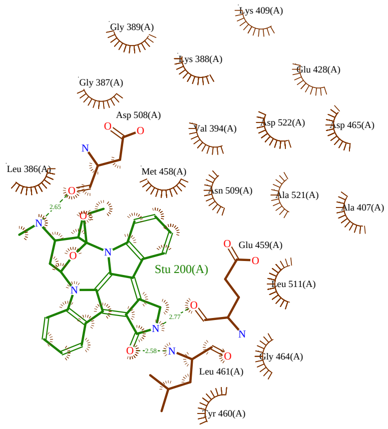

protein	amino acids contacts (binding site)
PDB:1xjd:chainA:Q04759	L G K G V A K E M E Y L G D D N L A D
sp:Q04759:KPCT_HUMAN	L G K G V A K E M E Y L G D D N L A D
tr:F1LM10:F1LM10_RAT	L G K G V A K E M E Y L G D D N L A D
tr:Q8CAV6:Q8CAV6_MOUSE	L G K G V A K E M E Y L G D D N L A D
sp:Q02111:KPCT_MOUSE	L G K G V A K E M E Y L G D D N L A D
tr:A6H667:A6H667_MOUSE	L G K G V A K E M E Y L G D D N L A D
sp:P83099:KPC4_DROME	L G K G V A K E M E Y L G D D N L A D
tr:B7Z147:B7Z147_DROME	L G K G V A K E M E Y L G D D N L A D
tr:Q9VYN1:Q9VYN1_DROME	L G K G V A K E M E Y L G D D N L A D
sp:P34722:KPC1_CAEEL	L G K G V A K E M E Y L G D D N L A D

	whole protein		domain-based		contact-based	
protein	ident	simil	ident	simil	ident	simil
PDB:1xjd:chainA:Q04759	1.0	1.0	1.0	1.0	1.0	1.0
sp:Q04759:KPCT_HUMAN	1.0	1.0	1.0	1.0	1.0	1.0
tr:F1LM10:F1LM10_RAT	0.94	0.98	0.97	0.99	1.0	1.0
tr:Q8CAV6:Q8CAV6_MOUSE	0.74	0.78	0.64	0.69	1.0	1.0
sp:Q02111:KPCT_MOUSE	0.95	0.98	0.96	0.99	1.0	1.0
tr:A6H667:A6H667_MOUSE	0.95	0.98	0.96	0.99	1.0	1.0
sp:P83099:KPC4_DROME	0.4	0.71	0.57	0.84	1.0	1.0
tr:B7Z147:B7Z147_DROME	0.4	0.71	0.57	0.84	1.0	1.0
tr:Q9VYN1:Q9VYN1_DROME	0.12	0.28	0.57	0.84	1.0	1.0
sp:P34722:KPC1_CAEEL	0.45	0.76	0.57	0.82	1.0	1.0

tpa-1 (WBGene00006599) associated phenotypes

antimicrobial gene expression variant, cell stress response variant, drug induced gene expression variant, isothermal tracking behavior variant, level of protein expression variant, phorbol ester resistant, response to injury variant

(Information from WormBase)

tpa-1 (UniProt:P34722) annotation

Function: Diacylglycerol (DAG)-dependent serine/threonine-protein kinase that phosphorylates a range of cellular proteins (By similarity). Phosphorylates mlk-1, a component of the JNK pathway. Involved in axon regeneration after injury probably by activating the JNK pathway (PubMed:23072806). (, PubMed:23072806).

(Information from UniProt)

Tyrosine-protein kinase ITK/TSK

Best gene implication in ageing for this target family came from gene P08630 annotated in UniProt release 2014_02. Annotation GO 8340 (determination of adult lifespan) was Inferred from Mutant Phenotype

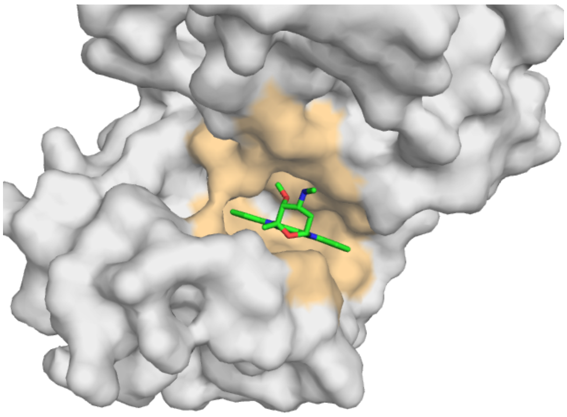

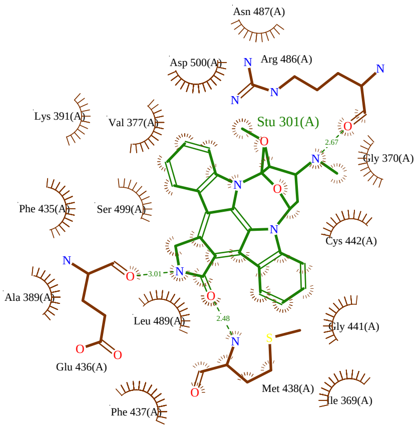

protein	amino acids contacts (binding site)
PDB:1sm2:chainA:Q08881	I G V A K F E F M G C R N L S D
PDB:1snu:chainA:Q08881	I G V A K F E F M G C R N L S D
sp:Q08881:ITK_HUMAN	I G V A K F E F M G C R N L S D
tr:D4A7W7:D4A7W7_RAT	I G V A K F E F M G C R N L S D
tr:Q8CAW3:Q8CAW3_MOUSE	I G V A K F E F M G C R N L S D
tr:A1A560:A1A560_MOUSE	I G V A K F E F M G C R N L S D
tr:Q5STT8:Q5STT8_MOUSE	I G V A K F E F M G C R N L S D
sp:Q03526:ITK_MOUSE	I G V A K F E F M G C R N L S D
sp:P08630:BTKL_DROME	L G V A K T E Y M G S R N L A D

	whole protein		domain-based		contact-based	
protein	ident	simil	ident	simil	ident	simil
PDB:1sm2:chainA:Q08881	1.0	1.0	1.0	1.0	1.0	1.0
PDB:1snu:chainA:Q08881	1.0	1.0	1.0	1.0	1.0	1.0
sp:Q08881:ITK_HUMAN	1.0	1.0	1.0	1.0	1.0	1.0
tr:D4A7W7:D4A7W7_RAT	0.94	0.97	0.97	0.99	1.0	1.0
tr:Q8CAW3:Q8CAW3_MOUSE	0.53	0.55	0.97	0.99	1.0	1.0
tr:A1A560:A1A560_MOUSE	0.94	0.98	0.97	0.99	1.0	1.0
tr:Q5STT8:Q5STT8_MOUSE	0.94	0.98	0.97	0.99	1.0	1.0
sp:Q03526:ITK_MOUSE	0.93	0.97	0.97	0.99	1.0	1.0
sp:P08630:BTKL_DROME	0.35	0.62	0.55	0.85	0.69	0.8

Btk29A (FBgn0003502) associated phenotypes

cell shape defective, lethal - all die before end of first instar larval stage, mating defective, partially, short lived, size defective, some die during first instar larval stage

(Information from FlyBase)

Btk29A (UniProt:P08630) annotation

Function: Required for proper ring canal development. Also required for the development of male genitalia and for adult survival. (PubMed:10330180, PubMed:9655810).
Cofactor: Zn(2+)Note=Binds 1 zinc ion per subunit. ;
Subcellular location: Note=Ring canals.
Tissue specificity: Ring canals in the egg chambers and imaginal disks of third-instar larvae. (PubMed:10330180, PubMed:3110602, PubMed:9655810).
Developmental stage: Expressed both maternally and zygotically. Predominantly in early to middle embryogenesis, in larvae and adult females. (PubMed:10330180, PubMed:3110602).
Disruption phenotype: Flies exhibit shortened copulatory duration (due to incomplete fusion of the left and right halves of the apodeme that holds the penis during copulation) and reduced adult-stage life span. (PubMed:10330180).

(Information from UniProt)

Ribosomal protein S6 kinase alpha-1

Best gene implication in ageing for this target family came from gene Q86NP0 annotated in UniProt release 2014_02. Annotation GO 8340 (determination of adult lifespan) was Inferred from Mutant Phenotype

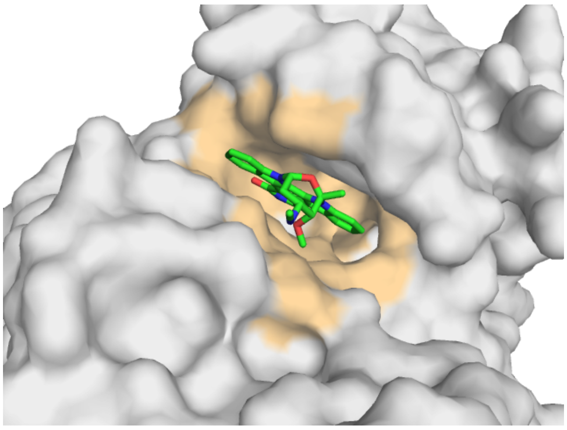

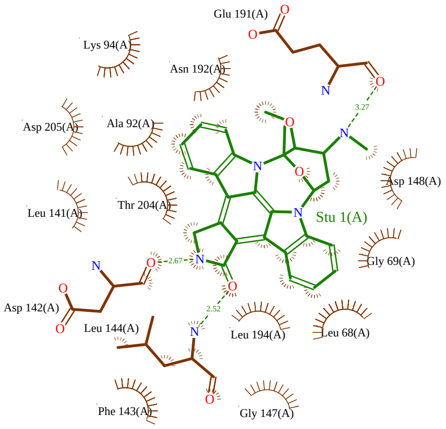

protein	amino acids contacts (binding site)
PDB:2z7r:chainA:Q15418	L G A K L D F L G D E N L T D
tr:B1AXG1:B1AXG1_HUMAN	L G A K L D F L G D E N L T D
tr:B7Z2K7:B7Z2K7_HUMAN	L G A K L D F L G D E N L T D
tr:B7ZB17:B7ZB17_HUMAN	L G A K L D F L G D E N L T D
tr:B4DG22:B4DG22_HUMAN	L G A K L D F L G D E N L T D
tr:E9PRI4:E9PRI4_HUMAN	L G A K L D F L G D E N L T D
tr:F5GYC4:F5GYC4_HUMAN	L G A K L D F L G D E N L T D
tr:E9PGT3:E9PGT3_HUMAN	L G A K L D F L G D E N L T D
sp:Q15418:KS6A1_HUMAN	L G A K L D F L G D E N L T D
sp:Q15349:KS6A2_HUMAN	L G A K L D F L G D E N L T D
tr:B7ZL90:B7ZL90_HUMAN	L G A K L D F L G D E N L T D
sp:P51812:KS6A3_HUMAN	L G A K L D F L G D E N L T D
sp:Q9UK32:KS6A6_HUMAN	L G A K L D F L G D E N L T D
tr:F2Z2J1:F2Z2J1_HUMAN	L G A K L D F L G D E N L T D
tr:F1M7N7:F1M7N7_RAT	L G A K L D F L G D E N L T D
tr:F1LXV0:F1LXV0_RAT	L G A K L D F L G D E N L T D
tr:D3Z8E0:D3Z8E0_RAT	L G A K L D F L G D E N L T D
tr:F1LYL8:F1LYL8_RAT	L G A K L D F L G D E N L T D
tr:B1AXP0:B1AXP0_MOUSE	L G A K L D F L G D E N L T D
tr:Q810Z3:Q810Z3_MOUSE	L G A K L D F L G D E N L T D
tr:B1AXN9:B1AXN9_MOUSE	L G A K L D F L G D E N L T D
tr:E9PWV3:E9PWV3_MOUSE	L G A K L D F L G D E N L T D
sp:P18653:KS6A1_MOUSE	L G A K L D F L G D E N L T D
sp:Q9WUT3:KS6A2_MOUSE	L G A K L D F L G D E N L T D
tr:F6RQA2:F6RQA2_MOUSE	L G A K L D F L G D E N L T D
tr:A2CEE6:A2CEE6_MOUSE	L G A K L D F L G D E N L T D
tr:Q505N6:Q505N6_MOUSE	L G A K L D F L G D E N L T D
tr:F6Q8A4:F6Q8A4_MOUSE	L G A K L D F L G D E N L T D
sp:P18654:KS6A3_MOUSE	L G A K L D F L G D E N L T D
tr:A2CEE5:A2CEE5_MOUSE	L G A K L D F L G D E N L T D
tr:Q810V8:Q810V8_MOUSE	L G A K L D F L G D E N L T D
tr:E9PUP0:E9PUP0_MOUSE	L G A K L D F L G D E N L T D
tr:A2CEE7:A2CEE7_MOUSE	L G A K L D F L G D E N L T D
tr:E9QK39:E9QK39_MOUSE	L G A K L D F L G D E N L T D
tr:Q86NP0:Q86NP0_DROME	L G A K L D F L G D E N L T D
tr:Q9VR61:Q9VR61_DROME	L G A K L D F L G D E N L T D
tr:Q24496:Q24496_DROME	L G A K L D F L G D E N L T D
sp:Q21734:KS6A1_CAEEL	L G A K L D F L G D E N L T D
tr:G5EE78:G5EE78_CAEEL	L G A K L D F L G D E N L T D

protein	whole protein ident	simil	domain-based ident	simil	contact-based ident	simil
PDB:2z7r:chainA:Q15418	1.0	1.0	1.0	1.0	1.0	1.0
tr:B1AXG1:B1AXG1_HUMAN	0.25	0.28	0.65	0.7	1.0	1.0
tr:B7Z2K7:B7Z2K7_HUMAN	0.79	0.8	1.0	1.0	1.0	1.0
tr:B7ZB17:B7ZB17_HUMAN	0.79	0.91	0.9	0.97	1.0	1.0
tr:B4DG22:B4DG22_HUMAN	0.79	0.91	0.9	0.97	1.0	1.0
tr:E9PRI4:E9PRI4_HUMAN	0.96	0.97	1.0	1.0	1.0	1.0
tr:F5GYC4:F5GYC4_HUMAN	0.78	0.89	0.9	0.97	1.0	1.0
tr:E9PGT3:E9PGT3_HUMAN	0.98	0.98	0.96	0.96	1.0	1.0
sp:Q15418:KS6A1_HUMAN	1.0	1.0	1.0	1.0	1.0	1.0
sp:Q15349:KS6A2_HUMAN	0.77	0.92	0.89	0.97	1.0	1.0
tr:B7ZL90:B7ZL90_HUMAN	0.71	0.89	0.86	0.96	1.0	1.0
sp:P51812:KS6A3_HUMAN	0.76	0.88	0.9	0.97	1.0	1.0
sp:Q9UK32:KS6A6_HUMAN	0.71	0.88	0.86	0.96	1.0	1.0
tr:F2Z2J1:F2Z2J1_HUMAN	0.75	0.89	0.89	0.97	1.0	1.0
tr:F1M7N7:F1M7N7_RAT	0.77	0.92	0.89	0.97	1.0	1.0
tr:F1LXV0:F1LXV0_RAT	0.98	0.99	0.99	1.0	1.0	1.0
tr:D3Z8E0:D3Z8E0_RAT	0.77	0.88	0.9	0.97	1.0	1.0
tr:F1LYL8:F1LYL8_RAT	0.6	0.77	0.86	0.96	1.0	1.0
tr:B1AXP0:B1AXP0_MOUSE	0.24	0.26	0.6	0.64	1.0	1.0
tr:Q810Z3:Q810Z3_MOUSE	0.5	0.57	0.89	0.97	1.0	1.0
tr:B1AXN9:B1AXN9_MOUSE	0.79	0.91	0.9	0.97	1.0	1.0
tr:E9PWV3:E9PWV3_MOUSE	0.94	0.96	0.99	1.0	1.0	1.0
sp:P18653:KS6A1_MOUSE	0.97	0.98	0.95	0.96	1.0	1.0
sp:Q9WUT3:KS6A2_MOUSE	0.77	0.92	0.89	0.97	1.0	1.0
tr:F6RQA2:F6RQA2_MOUSE	0.93	0.96	0.99	1.0	1.0	1.0
tr:A2CEE6:A2CEE6_MOUSE	0.63	0.79	0.86	0.95	1.0	1.0
tr:Q505N6:Q505N6_MOUSE	0.98	1.0	0.99	1.0	1.0	1.0
tr:F6Q8A4:F6Q8A4_MOUSE	0.93	0.96	0.99	1.0	1.0	1.0
sp:P18654:KS6A3_MOUSE	0.77	0.88	0.9	0.97	1.0	1.0
tr:A2CEE5:A2CEE5_MOUSE	0.67	0.85	0.86	0.95	1.0	1.0
tr:Q810V8:Q810V8_MOUSE	0.94	0.95	0.99	1.0	1.0	1.0
tr:E9PUP0:E9PUP0_MOUSE	0.65	0.83	0.86	0.95	1.0	1.0
tr:A2CEE7:A2CEE7_MOUSE	0.66	0.84	0.86	0.95	1.0	1.0
tr:E9QK39:E9QK39_MOUSE	0.6	0.76	0.86	0.95	1.0	1.0
tr:Q86NP0:Q86NP0_DROME	0.45	0.67	0.76	0.92	1.0	1.0
tr:Q9VR61:Q9VR61_DROME	0.46	0.68	0.76	0.92	1.0	1.0
tr:Q24496:Q24496_DROME	0.46	0.68	0.76	0.92	1.0	1.0
sp:Q21734:KS6A1_CAEEL	0.55	0.8	0.82	0.94	1.0	1.0
tr:G5EE78:G5EE78_CAEEL	0.54	0.78	0.82	0.94	1.0	1.0

S6kII (FBgn0262866) associated phenotypes
dominant, learning defective, male limited, memory defective, neuroanatomy defective, reduced, short lived

(Information from FlyBase)

rskn-1 (UniProt:Q21734) annotation
Function: Serine/threonine kinase that may play a role in mediating the growth-factor and stress induced activation of transcription.
Cofactor:Mg(2+)
Enzyme regulation: Activated by multiple phosphorylations on threonine and serine residues.
Ptm: Autophosphorylated on Ser-422, as part of the activation process.

(Information from UniProt)

rskn-1 (UniProt:G5EE78) annotation
Cofactor: Mg(2+) Evidence=(PIRNR:PIRNR000606);

(Information from UniProt)

Mitogen-activated protein kinase kinase kinase 5

Best gene implication in ageing for this target family came from gene P23561 via mapping the annotation from Ensembl YLR362W via mapping the annotation from EntrezGene 851076 via mapping the annotation from GenAgeModels 1609 annotated in GenAge release 17.

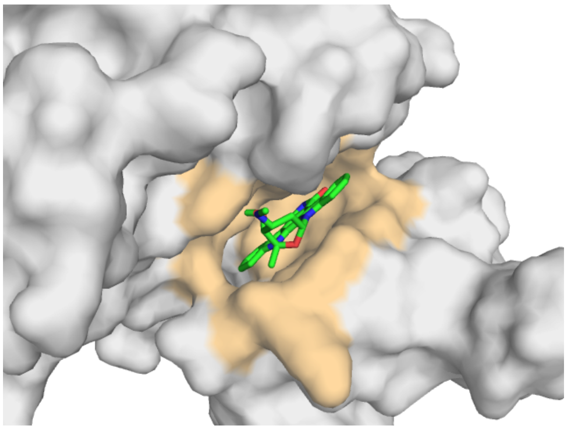

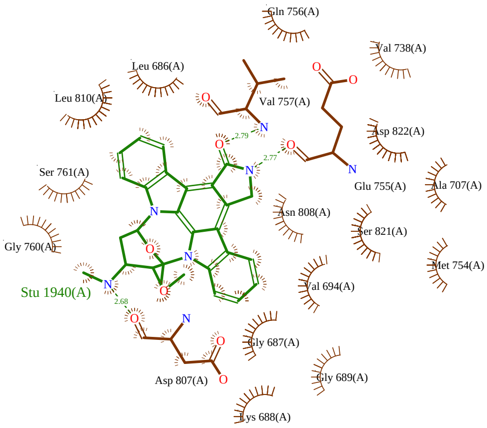

protein	amino acids contacts (binding site)
PDB:2clq:chainA:Q99683	L G K G V A V M E Q V G G S D N L S D
PDB:4bf2:chainA:Q99683	L G K G V A V M E Q V G G S D N L S D
sp:Q99683:M3K5_HUMAN	L G K G V A V M E Q V G G S D N L S D
tr:D3ZW27:D3ZW27_RAT	L G K G V A V M E Q V G G S D N L S D
tr:D3ZZH6:D3ZZH6_RAT	L G K G V A V M E Q V G G S D N L S D
tr:E9PWG9:E9PWG9_MOUSE	L G K G V A V M E Q V G G S D N L S D
sp:O35099:M3K5_MOUSE	L G K G V A V M E Q V G G S D N L S D
tr:Q24458:Q24458_DROME	L G K G V A V M E Q V G G S D N L S D
tr:Q7KSB7:Q7KSB7_DROME	L G K G V A V M E Q V G G S D N L S D
tr:Q8I6M5:Q8I6M5_DROME	L G K G V A V M E Q V G G S D N L S D
tr:Q9VDS9:Q9VDS9_DROME	L G K G V A V M E Q V G G S D N L S D
tr:Q4QQD0:Q4QQD0_DROME	L G K G V A V M E Q V G G S D N L S D
tr:B9EQQ2:B9EQQ2_DROME	L G K G V A V M E Q V G G S D N L S D
tr:Q21029:Q21029_CAEEL	L G K G V V V M E H V G G S D N L S D
sp:P23561:STE11_YEAST	I G S G V A V L E Y V G G S A N L T D

protein	whole protein ident	whole protein simil	domain-based ident	domain-based simil	contact-based ident	contact-based simil
PDB:2clq:chainA:Q99683	1.0	1.0	1.0	1.0	1.0	1.0
PDB:4bf2:chainA:Q99683	1.0	1.0	1.0	1.0	1.0	1.0
sp:Q99683:M3K5_HUMAN	1.0	1.0	1.0	1.0	1.0	1.0
tr:D3ZW27:D3ZW27_RAT	0.91	0.96	1.0	1.0	1.0	1.0
tr:D3ZZH6:D3ZZH6_RAT	0.92	0.97	1.0	1.0	1.0	1.0
tr:E9PWG9:E9PWG9_MOUSE	0.94	0.97	1.0	1.0	1.0	1.0
sp:O35099:M3K5_MOUSE	0.94	0.98	1.0	1.0	1.0	1.0
tr:Q24458:Q24458_DROME	0.22	0.38	0.73	0.91	1.0	1.0
tr:Q7KSB7:Q7KSB7_DROME	0.33	0.59	0.73	0.91	1.0	1.0
tr:Q8I6M5:Q8I6M5_DROME	0.36	0.68	0.73	0.91	1.0	1.0
tr:Q9VDS9:Q9VDS9_DROME	0.36	0.68	0.73	0.91	1.0	1.0
tr:Q4QQD0:Q4QQD0_DROME	0.36	0.68	0.72	0.91	1.0	1.0
tr:B9EQQ2:B9EQQ2_DROME	0.36	0.68	0.73	0.91	1.0	1.0
tr:Q21029:Q21029_CAEEL	0.31	0.65	0.62	0.85	0.89	0.95
sp:P23561:STE11_YEAST	0.1	0.28	0.32	0.62	0.68	0.78

Pk92B (FBgn0014006) associated phenotypes

heat sensitive

(Information from FlyBase)

nsy-1 (WBGene00003822) associated phenotypes

aldicarb hypersensitive, anoxia resistant, benzaldehyde chemotaxis variant, butanone chemotaxis variant, cell stress response variant, egg laying defective, egg laying response to food variant, gene expression level reduced, genetic pathway activation variant, lethal, levamisole hypersensitive, loss of asymmetry AWC, odorant adaptation variant, organism high sodium chloride hypersensitive, organism stress response variant, pathogen induced gene expression variant, pathogen susceptibility increased, pentanedione chemotaxis variant, protein phosphorylation reduced, pyrazine chemotaxis variant, sterile, transgene expression reduced

(Information from WormBase)
